# Exploring doctors’ perspectives on precision medicine and AI in colorectal cancer: opportunities and challenges for the doctor-patient relationship

**DOI:** 10.1186/s12911-025-03134-0

**Published:** 2025-07-30

**Authors:** Mirko Ancillotti, Åsa Grauman, Jorien Veldwijk, Åsmund Flobak, Deborah Mascalzoni

**Affiliations:** 1 Centre for Research Ethics and Bioethics, Department of Public Health and Caring Sciences, Uppsala University, Uppsala, Sweden; 2 Erasmus School of Health Policy & Management, Erasmus University Rotterdam, Rotterdam, The Netherlands; 3 Norwegian University of Science and Technology, Trondheim, Norway; 4 The Cancer Clinic, St Olavs Hospital, Trondheim, Norway; 5 Department of Biotechnology and Nanomedicine, Sintef Industry, Trondheim, Norway; 6 Institute for Biomedicine, Eurac Research, Bolzano, Italy

**Keywords:** Precision medicine, Artificial intelligence, Colorectal cancer, Doctor-patient relationship, Clinical decision-making, Medical ethics

## Abstract

**Background:**

Precision medicine and artificial intelligence (AI) are increasingly integrated into colorectal cancer (CRC) care, offering personalised treatment strategies and data-driven decision support. While these technologies promise improved outcomes, they also raise challenges concerning clinical decision-making, the doctor-patient relationship, and ethics. This study explores physicians’ perspectives on integrating precision medicine and AI in CRC care.

**Methods:**

A qualitative study was conducted using semi-structured interviews with ten CRC physicians from six European countries. Participants were recruited through purposive and snowball sampling. Interviews were analysed using thematic analysis.

**Results:**

Three key themes emerged from the analysis. First, physicians described precision medicine as a logical extension of existing tailoring practices, offering new opportunities while introducing complexity. Many expressed concerns about the blurred boundary between experimental and standard treatments, noting potential implications for equity and ethical decision-making. Second, AI was viewed as a future partner in care, with the potential to enhance efficiency and assist in synthesising complex data. However, participants voiced concerns about trust, clinical responsibility, and the lack of regulatory clarity, particularly due to AI’s “black box” nature. Finally, doctors reported challenges in communicating both precision medicine and AI-based recommendations to patients. They emphasised the importance of adapting communication strategies to individual patients and highlighted the need for structured approaches to ensure patient understanding and prevent miscommunication, especially when dealing with uncertain outcomes or emerging technologies.

**Conclusions:**

The findings highlight both the opportunities and challenges of integrating precision medicine and AI in CRC care. Addressing concerns related to communication, ethics, and regulation requires clear guidance and improved support for clinicians. Precision medicine and AI enhance CRC care but demand robust communication, regulation, and ethical safeguards to ensure transparency, trust, and physician autonomy.

**Supplementary Information:**

The online version contains supplementary material available at 10.1186/s12911-025-03134-0.

## Background

Precision medicine has emerged as a promising approach to address the limitations of traditional treatments by tailoring therapies based on the genetic, molecular, and environmental profiles of individual patients [1]. This approach has demonstrated potential in identifying actionable targets, optimising therapeutic outcomes, and minimising adverse effects. According to the World Health Organization, colorectal cancer (CRC) is the third most commonly diagnosed cancer and the second leading cause of cancer-related deaths globally, with an estimated 1.9 million new cases and 935,000 deaths in 2022 [2]. Standard treatments include chemotherapy, targeted inhibitors, and immunotherapy, the selection of which is based mostly on empirically founded clinical guidelines, and with some decisions based on genetic aberrations. For the majority of patients, chemotherapy is the main treatment [3, 4]. While these approaches have improved median survival for metastatic CRC (mCRC) patients to 30 months, resistance to therapy and relapse remain major challenges [5]. Advancing technologies such as next-generation sequencing, patient-derived organoids, and heavy computational techniques have further enhanced precision medicine strategies, paving the way for more personalised and effective care for CRC patients [6, 7]. Precision medicine is first and foremost relevant to metastatic disease since surgery provides curative strategies for most patients with localised disease, and for patients in a metastatic setting that exhaust standard treatment options, experimental approaches that integrate individualised research data into treatment can be considered [8]. The inter-patient genetic heterogeneity of tumours results in small subgroups, affecting traditional clinical trials by significantly reducing the number of eligible participants [9]. At the same time, precision medicine is transforming the role of doctors, requiring the integration of advanced computational tools, such as AI and machine learning, into clinical practice. AI is already being used in CRC care for tasks such as tumour detection, pathology evaluation, and treatment planning [10]. For instance, AI-based analyses of next-generation sequencing data can detect rare genetic mutations and identify novel therapeutic targets with greater speed and accuracy than traditional methods [7]. Recent research also highlights the potential for combining multi-omics approaches with AI to further enhance precision strategies, although their clinical implementation remains limited [11]. These developments underscore the growing presence of AI in CRC treatment decision-making [12]. Consequently, oncologists and other health professionals involved in CRC care face the challenges of developing competencies in interpreting complex molecular data and collaborating with multidisciplinary teams, such as molecular tumour boards, to make informed decisions [13].

Despite these rapid developments, there is limited understanding of how precision medicine and AI are shaping the clinical roles, responsibilities, and decision-making practices of physicians. Ethical, communicative, and relational implications, particularly those concerning trust and patient involvement, have received relatively little empirical attention. This study contributes to addressing this gap by exploring how physicians working with CRC experience and respond to the opportunities and challenges brought by these technologies.

The integration of precision medicine and computational tools also reshapes the doctor-patient relationship, introducing both opportunities and challenges. As highlighted by Grauman et al. [14], on the one hand, precision medicine offers highly individualised care, potentially enhancing patients’ trust in their doctors who show a commitment to tailoring treatment to their specific needs. On the other hand, reliance on AI and data-based systems may create barriers to communication, as patients may struggle to understand the rationale behind algorithmic recommendations. Shared decision-making, a cornerstone of the deliberative doctor-patient relationship model, becomes more complex in this context, as doctors must explain intricate molecular findings while respecting patient autonomy [14]. Additionally, they must communicate the inherent uncertainty in these data-based approaches—an aspect that is neither trivial to articulate nor easy for patients to absorb. Precision medicine raises ethical concerns about trust, responsibility, and balancing human judgment and machine-based recommendations. Introducing AI-based decision support systems may shift aspects of clinical responsibility from physicians to computational models, raising questions about liability in cases of error or adverse outcomes [14, 15]. Moreover, patients’ trust in their oncologists could be undermined if AI systems are perceived as opaque or the reasoning behind treatment choices is inadequately explained [14, 16].

Against this background, this study aims to explore doctors’ perspectives on precision medicine and the integration of heavy computational methods in CRC. It focuses on their views regarding the implications of precision medicine and AI for clinical decision-making, the doctor-patient relationship, and ethical considerations related to trust, communication, and responsibility. By examining these perspectives, the study seeks to identify potential opportunities and challenges associated with precision medicine in CRC. There is limited empirical research on how practising physicians perceive the integration of precision medicine and AI in CRC care, particularly regarding decision-making and the doctor-patient relationship. As highlighted in a recent systematic review, such perspectives remain underexplored [14]. This study addresses this gap by examining how CRC physicians interpret the opportunities and challenges posed by these technologies in clinical practice.

## Methods

### Study design and interview guide

To gain in-depth insights into the opinions and experiences of physicians involved in the care of CRC patients, a qualitative inductive design was applied, using an emerging design approach [17]. This allowed for flexibility both in response to recruitment challenges, combining purposive and snowball sampling, and in conducting the interviews, which followed a semi-structured guide that could be adapted to each participant’s background and responses.

A semi-structured interview guide with open-ended questions was developed based on a literature review on the impact of precision medicine on the doctor-patient relationship, highlighting areas of concern such as doctor-patient communication, decision-making in a trusting relationship, and tensions connected to conventional and experimental treatments [14]. To refine the interview guide, multiple meetings were held with researchers from different backgrounds, including computational biology, systems biology, clinical oncology, computational modelling, and ethics, within the project consortium. This preparatory work led to the identification of five areas of interest, namely, precision medicine conceptualisation, doctor-patient communication, attitude towards computational techniques, trust issues connected to precision medicine and computational techniques, and responsibility issues connected to precision medicine and computational techniques (see Appendix 1 for the full interview guide). A mapping of the interview questions to the main research focus areas is provided in Appendix 2.

### Recruitment of participants

**Fig. 1:**
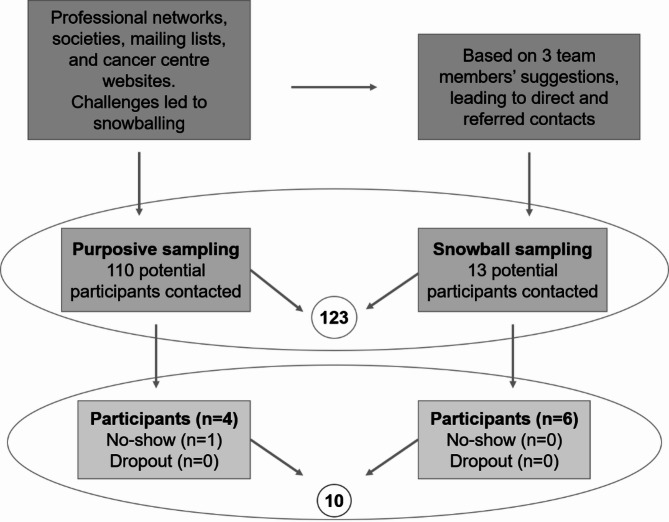
Recruitment process

Participants were recruited through a combination of purposive and snowball sampling [18]. The inclusion criteria for participating were having a pertinent specialisation, working with CRC patients in a European country, and being English-speaking. Geographic variation was pursued. Initial recruitment efforts included reaching out via professional networks, medical societies, conference mailing lists, and publicly available contact information on cancer centre websites. While these strategies led to some successful contacts, recruitment challenges emerged, prompting the integration of snowball sampling (see Fig. 1). This approach not only supported recruitment continuity but also contributed to increasing diversity in terms of geography and professional background. Participants were initially contacted through email, and those interested were provided with a research information sheet. After addressing any questions, interviews were scheduled.

### Data collection

The interviews were conducted online through the Zoom platform between May 2023 and January 2024. The interviews were conducted in English, audio-recorded and transcribed verbatim by a professional transcription service. Interview excerpts have been lightly edited for readability while preserving participants’ intended meaning. The notion of “information power” was employed to determine when to stop recruitment and data collection [19]. The notion of information power suggests that sample adequacy depends not on numbers alone, but on the relevance, specificity, and richness of the information provided by participants in relation to the study aim. This involved multiple meetings where members of the research team discussed whether the data collected was sufficiently relevant and detailed to answer the research aim and whether data saturation was expected; these discussions also included practical considerations (like time constraints and resource limitations).

### Data analysis

Transcripts were analysed using thematic analysis with a constructionist frame and experiential orientation [20]. Thematic analysis was selected as a method suitable for identifying recurring patterns in the data. A constructionist frame and experiential orientation were employed to examine how participants made sense of their experiences, considering how their views were shaped by broader institutional and professional discourses related to AI and precision medicine [21]. The analysis was conducted with an inductive approach, using semantic and latent coding. The process included the following steps: immersive reading and familiarisation with the data; generation of initial codes; generation of themes; review and definition; production of the report. Two independent researchers were involved in the full analysis (MA & ÅG). First, they independently analysed the same sample of two interviews and discussed and compared their coding and preliminary themes. Afterwards, MA independently analysed the remaining data, holding regular meetings to discuss their impressions and reflections with the rest of the full research team, whose expertise includes clinical oncology, public health, ethics, and patient preferences. The software used for data management during the analysis was Microsoft Office Excel 2007. The interview transcripts were submitted to the attention of the interviewees, who were encouraged to contact the research team to make any necessary changes. One independent researcher with a background in medical anthropology, global health, and medical biotechnology was involved in an audit trail towards the end of the study [22].

## Results

Ten physicians specialised in oncology and gastroenterology (seven men and three women) from six European countries (France, Italy, the Netherlands, Norway, Spain, and Sweden) were interviewed. The participants’ mean age was 52 years (min-max range 35–77), and they had an average of 20 years of experience in the care of CRC patients (min-max range 10–45). All of them combined research with their clinical work. The mean interview duration was 26 min (min-max range 14–37).

### Themes

**Fig. 2:**
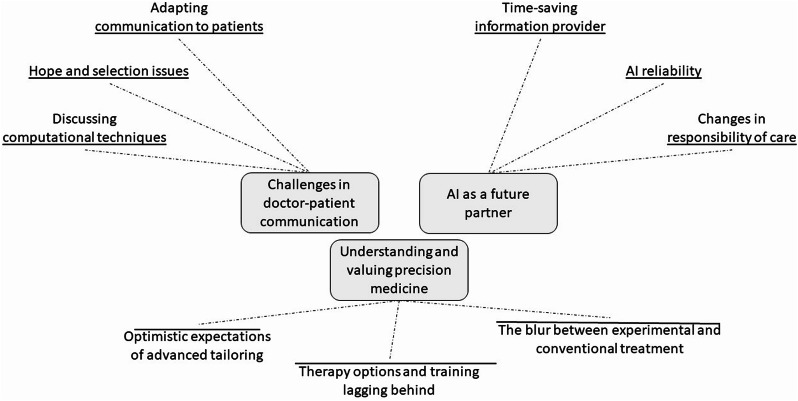
Themes and sub-themes obtained in the analysis

The analysis yielded three themes with additional sub-themes (see Fig. 2): *New technology builds on previous tailoring*,* AI as a future partner*,* and Challenges in doctor-patient communication*. Tables 1, 2 and 3 contain quotations from the interviews organised by theme with the purpose of illustrating the analysis process and findings through examples [23].

#### Understanding and valuing precision medicine

This theme reports participants’ interpretations of precision medicine and their reflections on experimental and ordinary treatments.

##### Optimistic expectations of advanced tailoring

For the majority, precision medicine was considered the future of oncology, enabling treatment options that could avoid unnecessary side effects and spare lives (Table 1, Q1). Although there was some indecision about the difference between precision medicine and personalised medicine, for the most part, the former was described in terms of tailoring the treatment to the characteristics of the person and the tumour (Table 1, Q2). Since using different biomarkers to guide treatment decisions is not new, some participants rejected the hype around precision medicine. The novelty is the increased number of biomarkers that enable even more complicated decisions (Table 1, Q3).

##### Therapy options and training lagging behind

Inquired about the negative sides of precision medicine, participants mentioned the difficulty of staying updated. One participant stated that even though gathering a lot of information from large-scale gene panel analyses is possible, there is a lack of targeted therapies to offer the patients (Table 1, Q4).

##### The blur between experimental and conventional treatment

In the discussion of ordinary and experimental treatment options, participants emphasised the scarcity of treatment options in CRC and information about mutations as a stimulus to test and find innovative ways of treating the patients, somehow blurring the threshold between ordinary and experimental (Table 1, Q5). Not everybody felt comfortable with providing experimental treatment, raising issues of treatment uncertainty and care standards, but also equality in healthcare, as for patients who can afford it, there are private hospitals that offer experimental treatments (Table 1, Q6).

**Table 1:** Exemplar quotations of the theme ‘Understanding and valuing precision medicine’

Number	Excerpt
Q1 (Norway)	*…what I believe will be oncology in 20 years or so? Where we will hopefully be able to cure most of our patients*,* even though they have what today is considered incurable disease.*
Q2 (France)	*I think medicine is already precision in a way because we are always adapting our choices to the person we have in front of us. And then*,* on the disease—of course*,* in cancer—the most active part of precision medicine*,* which can be seen from multiple angles*,* in my opinion*,* remains the molecular assessment of the tumour to try to adapt as much as possible to the treatment of the specific molecular subtypes of each tumour.*
Q3 (Sweden)	*There is currently a hype around precision cancer medicine. That is a bit*,* I would say*,* well*,* not fully motivated. I mean*,* precision medicine is about using different biomarkers to guide us in making treatment decisions*,* and that is not something new. What has happened is that the technological development has allowed us to look for many more biomarkers*,* so that we could make*,* so to speak*,* more complicated decisions.*
Q4 (Norway)	*…all the ability to do more and more large-scale gene panel analysis and gathering a lot of information*,* but with the limitation of not having enough targeted therapies to use all this information*,* at least not in a therapeutic way. Thus*,* precision medicine still today is not the only solution for cancer patients. So*,* we still depend on the classical treatment options.*
Q5 (Norway)	…*in colorectal cancer*,* we have a very limited amount of treatment options*,* which we could kind of define as precision cancer medicine … And even though we do some single-gene analysis in colorectal cancer*,* that’s much more limited. And so*,* to my mind*,* it’s not upfront experimental anymore*,* but of course*,* the more sophisticated analysis you do*,* and the more*,* obviously*,* aberrations from the normal state you find*,* the more natural it is to look into. Okay*,* now we have found this mutation. Is there any treatment anywhere in any clinical trial which would fit for this specific mutation? So*,* it’s natural*,* I think*,* for the physician to much earlier and much easier think about experimental treatment when you have a lot of more sophisticated information*,* mainly being gene panel analysis. But for me*,* and in my conversations with patients*,* not every precision cancer treatment is necessarily experimental anymore. I don’t think so anymore.*
Q6 (Sweden)	*Then of course*,* should you give treatment outside of trial*,* being experimental? Well*,* basically not*,* but it is*,* it is given. There are private hospitals today or private departments that give this*,* and patients who can afford it go to wherever they can in the world and want that type of treatment.*

#### AI as a future partner

This theme encapsulates participants’ opinions on using AI and other heavy computational methods in different phases of CRC patients’ care.

##### Time-saving information provider

In general, participants placed their answers in the future and hypothetical scenarios. While some participants were not convinced by the use and future use of AI in CRC care, most regarded AI as a partner providing information, saving time and providing communication tools. Something considered especially useful to young doctors. (Table 2, Q7).

##### AI reliability

In general, the use of algorithms, AI, and other advanced computational methods was considered an inevitable development in CRC care, to which patients would gradually adapt. Some patients were expected to be especially open and enthusiastic about using AI. In contrast, others would be more conservative but may accept digital tools if the doctor trusts them (Table 2, Q8). The matter of trust in AI permeated the conversations. Doctors did not express open scepticism but wondered how to effectively rely on AI results (Table 2, Q9). Indeed, it was emphasised that beyond trust, the use of the tools by the doctors would matter (Table 2, Q10). Concerns were raised about whether AI may lead to privacy issues and if it could cause troublesome changes in the doctor-patient relationship (Table 2, Q11).

##### Changes in responsibility of care

Issues of doctors’ and other involved actors’ responsibility implied by AI and other heavy computational methods were discussed. Most participants agreed that the responsibility of care stays with the doctors and the team (Table 2, Q12). However, the notion that responsibility may be shared with AI developers and those who recommend or impose their use was also mentioned. Similarly, the concern was voiced that doctors in the future may feel or believe they are less responsible for the decisions made using AI tools (Table 2, Q13).

**Table 2:** Exemplar quotations of the theme ‘AI as a future partner’

Number	Excerpt
Q7 (Norway)	*So*,* what I would like to have is an AI tool into which you put all the information that you have. So*,* any gene panel analysis*,* as well as the patient’s comorbidities*,* drugs and everything you have information on. And ideally*,* this information wouldn’t have to be put into this AI tool by myself*,* but it is just automatically gathered from the patient’s electronic chart. Then the AI tool sorts out all the things I don’t need to care about*,* either because there is no relevance to it or because they simply don’t have anything to do with the problem I am expected to handle. And then it leaves me on the screen with a one-page summary of the important genomic aberrations*,* important comorbidities*,* and important other things to consider for this patient.*
Q8 (France)	*Doctors are very well trusted by the population in general*,* even if it’s getting a little worse every 10 years. But I think the relationship with a human being remains central to the way French people think. But it may change in the future. And I would say that today*,* if we were to propose these kinds of tools*,* probably*,* as usual*,* around 60 per cent of patients would follow what the doctor is saying. If we use the tool*,* they will use it and accept its guidance.*
Q9 (Netherlands)	*It really depends on what you do with AI. I can understand*,* for instance*,* that when you have a scan*,* and the scan is only seen by AI*,* and we fully rely on what it determines based on its algorithms*,* then I think this could create suspicion among patients. I think I would be suspicious as well*,* a bit*,* because we’re not sure whether this AI is good enough.*
Q10 (Netherlands)	*It depends on how you use the algorithm. So if you use the algorithm just as a tool*,* which helps the doctor and the patients to have more insights into what is maybe going to happen*,* then you can still decide to follow or not follow the suggestion.*
Q11 (Italy)	*Anyway*,* I’m afraid too*,* of such a way to do medicine because the risk we run is to be in front of a PC and to insert data*,* and then to inform the patient of what is the result of our software. So*,* the relationship between the doctor and patient could change in this way.*
Q12 (Netherlands)	*I think the doctor is still responsible. It’s our responsibility to decide whether the AI is good enough. So if it’s not good enough*,* we shouldn’t use it. And if we rely on it*,* it’s our responsibility that it also works as we want it to work. We have to check whether what AI says is also correct. I think you can’t rely on a company that provides the technology to us because they are not the doctor. So*,* it’s still our responsibility*,* I think.*
Q13 (France)	*I think*,* of course*,* for the doctors*,* it will be very difficult to feel as responsible as they are today with a bot intervening in the decision.*

#### Challenges in doctor-patient communication

This theme illustrates the challenges of doctor-patient communication in CRC as it emerged in discussing specific topics, such as properly informing the patients about the treatment, the distinction between experimental and ordinary treatment, the use of AI and other heavy computational methods, and concerns about trust and doctors’ responsibility. Participants emphasised the uniqueness of the patient-doctor relationship in oncology and the importance of building the relationship (Table 3, Q14).

##### Adapting communication to patients

The participants reflected that communication always must be adapted; some CRC specialists mentioned that some patients rely heavily on their judgment, while others want more explanations and involvement (Table 3, Q15). Patients can be very well informed, which may be detrimental at times, as attaining too much information could be confusing or expensive (Table 3, Q16). Overall, most participants agreed on the duty to adapt their communication to their patients’ needs, although it may be difficult to understand what the right amount of information is or how to explain details (Table 3, Q17).

##### Hope and selection issues

Communication difficulties in precision medicine were related to the uncertainty of the treatment, explaining technology, a cognitive bias in favour of ‘new’ treatment options and connected exaggerated expectations, as well as hope and selection issues typically connected to whether a patient was considered eligible or not for an experimental treatment (Table 3, Q18). For the most part, the provision of experimental treatment was considered a matter of informed consent and proper communication (Table 3, Q19).

##### Discussing computational techniques

Issues of trust implied by AI and other heavy computational methods were discussed regarding trust in the AI results and how using AI could affect trust in the doctor-patient relationship. Some participants reflected that the patient’s trust in doctors would influence their trust in AI tools. Although clear communication and the need to adapt were shared values, some participants stated that they did not discuss algorithms, AI, or other heavy computational methods with the patients, not even when AI tools in imaging techniques were used (Table 3, Q20). However, while discussing whether AI introduction implies changes in doctors’ responsibilities, some rejected the responsibility framing and considered it a communication matter (Table 3, Q21).

**Table 3:** Exemplar quotations of the theme ‘Challenges in doctor-patient communication’

Number	Excerpt
Q14 (Italy)	*It’s very hard to think about this*,* because the relationship between patient and doctor is not only about the decision of the treatment*,* but it’s a life together*,* some years of life together. So*,* I don’t think artificial intelligence could substitute all the aspects of this relationship.*
Q15 (France)	*Generally*,* I try to explain*,* but I can see that some patients are not interested and tell me: okay*,* I don’t care about that*,* this is not my topic*,* I was building houses*,* I don’t know anything about medicine. So*,* tell me what I have to do. Some patients are younger*,* more educated*,* or more of both and ask very detailed questions*,* and some patients are experts.*
Q16 (France)	*some patients who are very well informed are also trying to control a lot of what is going to happen. And sometimes they do not behave adequately for their health*,* I would say*,* because they try to get many opinions*,* they get confused*,* sometimes they make the wrong choices. They go to a private practice hospital with fantastic promises that will cost a lot but is not really worth it. So*,* it can be perverse.*
Q17 (Italy)	*Sometimes it’s difficult because we have to speak of a gene*,* of mutations*,* of targeted treatments. So*,* it’s not so simple to explain these problems to a patient who did not study medicine or molecular biology. So*,* we have to find the correct words to be clear*,* to be understandable.*
Q18 (Norway)	*And I think there is always a problem with cancer patients. You know*,* when I say*,* “I don’t want to give you that treatment because it probably won’t work*,*” they ask*,* “But is there a 1% chance that it might work?” And I say*,* “Yes*,* there is always a 1% chance that it might work*,*” and then they want it. So*,* it’s very difficult when the alternative is death.*
Q19 (Netherlands)	*I think the most important thing is that the patient understands the different treatment options*,* including precision treatment*,* experimental treatment*,* and conventional treatment. I do think that patients are able to make a decision together with the doctors*,* and sometimes*,* it’s the doctor who may help by guiding them to make a good decision.*
Q20 (Netherlands)	*But if you are fairly sure that it’s making treatment better*,* then I don’t think it’s needed to discuss it when it’s adding to our knowledge and to our ability to treat patients. You only need to discuss it when it’s going to alter your thinking*,* and your decision-making depends on AI*,* and you can make decisions that otherwise you wouldn’t have made that are potentially also harmful to the patient. Then I think you have to discuss it.*
Q21 (Netherlands)	*I don’t think anybody is to blame. If the data is robust*,* you get the sensitivity and specificity of a model*,* and then you know for sure that it will never be 100 per cent for both*,* right? I think this is something you have to discuss with your patient before starting treatment—that there’s always a chance it will not work or may not be the best option for that specific patient.*

## Discussion

This study explored physicians’ perspectives on integrating precision medicine and AI in CRC care, focusing on implications for clinical decision-making, the doctor-patient relationship, and associated ethical considerations. The findings highlight both opportunities and challenges posed by these advancements.

### Opportunities and limitations of precision medicine

Participants viewed precision medicine as a significant advancement, enabling tailored treatment strategies that enhance therapeutic outcomes and reduce adverse effects. The use of biomarkers and molecular profiling was particularly valued for its role in guiding treatment decisions and improving survival rates in mCRC. However, the increasing number of biomarkers also introduces complexities, as both participants and prior research suggest that these advancements can complicate clinical decision-making and contribute to scepticism by reinforcing the perception of ‘hype’ surrounding precision medicine [9, 24]. Some physicians noted that tailoring treatments is not a new concept, but the growing complexity of molecular profiling underscores both its potential and its challenges. Furthermore, the limited availability of targeted therapies for CRC underscores the gap between identifying actionable mutations and offering effective treatments [3, 25]. These limitations highlight the need for continued investment in therapeutic innovation to increase precision medicine benefits.

Another challenge is the increasingly blurred line between experimental and conventional treatments. Historically, oncology clinical trials assessed drugs for broad patient populations, but precision medicine has shifted the focus toward more individualised trial designs, including ‘N-of-1’ trials, where treatments are personalised based on a patient’s molecular, immunological, and biological characteristics [26]. Physicians in this study noted that, due to the scarcity of treatment options in CRC, experimental approaches are often considered, which raises concerns about equity and care standards. While equitable access to precision medicine should be prioritised, as advocated by the European Code of Cancer Practice [27], precision oncology may exacerbate disparities in cancer care, particularly when biomarker-driven treatments are not universally accessible [24]. Recent research has identified significant disparities in biomarker prevalence among different ancestry groups, which may affect eligibility for precision oncology therapies. For instance, patients of African ancestry were found to be less likely to have actionable biomarkers, potentially limiting their access to targeted treatments. This underscores the need for more inclusive research and consideration of diverse populations in developing precision medicine protocols [28]. Furthermore, traditional randomised controlled trials rely on equipoise, ensuring genuine uncertainty about treatment efficacy [29]. However, precision medicine often necessitates departures from this model. For instance, customised drug combinations tailored to individual patients sometimes rely on uncontrolled phase I trials rather than randomised designs [30]. While proponents argue that the expected benefits of novel drugs justify this shift, others warn of scientific and ethical concerns [30]. These findings underscore the need for clear guidelines, robust communication, and ethically defensible practices to ensure informed consent and protect patient interests.

### AI as a partner in CRC care

AI and computational tools were generally regarded as valuable assets in CRC care, particularly for enhancing efficiency, generating insights, and aiding less experienced physicians. Participants envisioned AI as a “future partner” capable of synthesising vast data and presenting actionable recommendations. AI applications in CRC span a broad spectrum, including detection, diagnosis, grading, characterisation, drug discovery, treatment, and prevention [7, 31]. Given the inherent heterogeneity of CRC, it can be a relevant model for applying precision medicine principles. This individualised approach, informed by AI-powered insights, has the potential to contribute to the development of more targeted treatment strategies [7, 31].

However, concerns about trust, responsibility, reliability, and privacy emerged. While some physicians expressed optimism about AI’s potential to gain patient acceptance, others emphasised the risk of undermining trust in the doctor-patient relationship if AI tools are perceived as opaque or overly dominant in decision-making. Patients’ trust in AI may largely depend on their trust in physicians, as patients often lack an understanding of how AI systems operate [14, 32]. In precision cancer medicine, uncertainty further amplifies the need for clear communication, as patients rely heavily on physicians for guidance [33].

The issue of responsibility in AI-assisted care was considered a central theme by the participants. Most participants agreed that the primary responsibility, for instance, for treatment decisions, should remain with physicians, although they acknowledged that developers and institutions deploying AI tools share accountability. The “black box” nature of AI complicates this distribution of responsibility, as clinicians may feel pressured to rely on AI-generated recommendations without fully understanding their rationale [15]. Regulatory challenges add another layer of complexity, particularly as AI continuously learns and updates, requiring mechanisms to ensure human oversight remains central [34]. Participants also expressed concerns about biases in AI systems, particularly given that healthcare AI applications depend on large datasets that may not represent diverse patient populations [35]. Without rigorous validation processes, there is a risk that AI could reinforce systemic biases in clinical decision-making. Diprose et al. explored how physicians viewed a hypothetical machine learning–based risk calculator and found concerns that are similar to those raised by participants in our study. These included difficulties understanding how the tool generated its output, uncertainty about who would be responsible for decisions based on its recommendations, and possible effects on the doctor-patient relationship [36]. Although the clinical context and technology differ, the findings highlight broader issues that clinicians encounter when attempting to integrate AI into care. In both cases, there was a clear need for greater transparency and better ways of explaining algorithmic support to patients.

Integrating AI into clinical care also raises significant privacy and data security concerns. As AI-based systems increasingly rely on large-scale health data, including genomic and behavioural information, traditional consent models may be insufficient. According to Shearer et al. [34], new frameworks for data stewardship and patient autonomy are needed to protect sensitive information while ensuring meaningful and informed patient engagement. Furthermore, if AI implementation is not accompanied by clear communication strategies that emphasise human oversight and shared decision-making, there is a risk that patients will perceive it as a depersonalising force in medicine [14].

The findings underscore the need for a cautious and well-regulated approach to AI adoption in healthcare. While AI offers the potential to enhance medical decision-making, its implementation must be carefully managed to preserve trust, ensure accountability, and safeguard patient rights.

### Challenges in doctor-patient communication

Effective communication was consistently identified as a cornerstone of integrating precision medicine and AI into CRC care. Physicians acknowledged the challenge of explaining complex concepts, such as gene mutations and AI-based recommendations, particularly to patients without a medical background. Striking the right balance between providing sufficient information and avoiding confusion remains difficult [37]. In CRC care, ineffective communication is a recognised factor that contributes to disparities in patient awareness and engagement [38].

The ethical dilemma of managing patients’ hopes and expectations was another key concern, especially regarding experimental treatments. While precision medicine and AI introduce new opportunities, they also risk fuelling unrealistic optimism [14], necessitating transparent and empathetic communication. If not properly contextualised, AI-based recommendations may lead patients to misinterpret their significance, further complicating the physician’s role in guiding treatment decisions [34].

Discussing the use of heavy computational methods in CRC care was a thorny issue for most participants. Triberti et al. warn of the potential “third wheel” effect, where AI’s involvement in medical decision-making may disrupt the traditional physician-patient relationship [16]. This disruption can manifest in three ways: delays in decision-making when AI recommendations are difficult to explain, misalignment between AI classifications and patient symptoms, and ambiguity in the roles and authority of healthcare providers [16]. Additionally, AI’s lack of human empathy and relational engagement may further contribute to patient hesitation in accepting AI-based medical guidance [39]. To prevent such disruptions, it is important that AI is interpreted at all times as a supportive tool rather than an autonomous decision-maker, ensuring transparency and reinforcing physician authority in patient care.

The study findings emphasise the importance of integrating AI and precision medicine in CRC care through clear communication strategies that enhance patient understanding and autonomy while ensuring that physicians remain central to medical decision-making, with their expertise and clinical judgment recognised as essential in the doctor-patient relationship.

### Limitations

Rather than focusing on sample size as a measure of rigour, the study design was guided by the concept of information power, ensuring that the data were sufficiently rich and relevant to address the research questions [19]. While the findings provide valuable insights, they reflect the experiences of a specific group of European physicians and may not be directly transferable to other healthcare settings or specialities. Additionally, participants may have been more likely to have a professional interest in precision medicine and AI, which could introduce selection bias and influence their perspectives on these topics. The decision to include only English-speaking participants was made to ensure consistency across interviews and reduce the risk of interpretive bias introduced by language variation. However, this may have limited the inclusion of some physicians who did not feel comfortable being interviewed in English, even if they met al.l other inclusion criteria. It may also have reduced representation from parts of Europe where English is less commonly used in professional settings. This limitation is partly inherent in the qualitative design, which prioritises contextual depth over statistical generalisability. Compared to survey-based or scenario-driven studies, the qualitative approach employed here facilitated a more detailed and context-sensitive understanding of how physicians interpret and navigate the clinical, ethical, communicative, and professional dimensions of precision medicine and AI.

Multiple strategies were employed throughout the research process to enhance trustworthiness. Researcher triangulation allowed for a reflexive approach to data interpretation, while peer debriefings helped challenge assumptions and refine thematic coding. Additionally, member checking ensured that participants’ views were accurately represented [20, 22]. However, findings are context-dependent, and future research could expand on these insights by incorporating patient perspectives and examining the longitudinal effects of precision medicine and AI integration in clinical practice.

## Conclusion

The physicians interviewed in this study regarded precision medicine as contributing to treatment personalisation and improved treatment outcomes. However, its growing complexity, the limited availability of targeted therapies, and the blurred distinction between experimental and conventional treatments were seen as obstacles to its effective clinical implementation and equitable patient access. AI was recognised as a valuable partner in CRC care, particularly for data synthesis and decision support, but concerns regarding trust, responsibility, and regulatory oversight were voiced. Physicians expressed worry about AI’s black-box nature, potential biases, and the redistribution of clinical authority and responsibility. A key issue identified was the challenge of doctor-patient communication in the context of precision medicine and AI. Physicians emphasised the difficulty of conveying complex genetic and AI-based recommendations to patients. There was a shared view that structured communication strategies are essential for maintaining patient trust and supporting informed decision-making. The findings suggest that the successful integration of AI and precision medicine in CRC care will require clearer regulatory frameworks, ethical safeguards, and support for clinicians’ communicative responsibilities. Ensuring that AI remains a supportive tool rather than an autonomous decision-maker is crucial for maintaining patient trust and physician autonomy. Future research should explore patient perspectives and investigate how these technologies impact long-term clinical outcomes and decision-making processes. While this study focused on CRC care, some of the findings may be relevant to other forms of cancer. However, further research is needed to assess the extent to which these perspectives apply to different tumour types and clinical contexts.

## Supplementary Information

Below is the link to the electronic supplementary material.

Supplementary Material 1

## Data Availability

The interviews analysed during the current study are available from the corresponding author on reasonable request.

## Abbreviations

AI: Artificial Intelligence
CRC: Colorectal Cancer
mCRC: Metastatic Colorectal Cancer
