# Supplementary material for: Exploring doctors’ perspectives on precision medicine and AI in colorectal cancer: opportunities and challenges for the doctor-patient relationship
Source: BMC Med Inform Decis Mak. 2025 Jul 30;25:283. doi: 10.1186/s12911-025-03134-0 (PMC12312572; doi:10.1186/s12911-025-03134-0)
Supplement: Supplementary file 1 — Supplementary Material 1 [file 12911_2025_3134_MOESM1_ESM.docx]

# Appendices

Appendix 1: Interview protocol

Appendix 2: Mapping research focus to interview questions

## Appendix 1

### Interview protocol

My name is Mirko Ancillotti, and I am a researcher at Uppsala University.

This study is being conducted as part of a large European project called OncoLogics. This project aims to build a system that helps doctors make better treatment decisions for patients with colorectal cancer. Within this project, my team and I examine the ethical, legal, and social aspects of medicine and technology. Indeed, the main aim of this interview is to study your views about precision medicine, and computer-based decision aids, as well as what you think patients expect from these developments.

You have been invited to this interview since you are a stakeholder.

With the information we get from these interviews, we will publish an article in which we report on the viewpoint of European doctors, and we will also draft a survey for further investigation.

The interview shouldn’t last more than 45 minutes. First, I will ask you for some background information, and then we will start our conversation. Please consider that participation is voluntary and that you can pause or withdraw at any point.

I will record this interview, and only the audio track will be kept. The track will be transcribed, and any detail that could potentially lead to the identification of the interviewee or third parties will be removed. Recording and transcripts will be stored in a secure archive at Uppsala University and kept for ten years. You can find all the details in the ‘Participant Information’ document that I sent you.

Do you want to participate in this interview?

### Background information

1. What year were you born?

□ _ _ _ _

2. Are you □ Woman

□ Man

□ Other

3. What is your specialisation?

4. How many years of experience do you have in taking care of colorectal cancer patients?

5. Do you combine your clinical work with research?

Before starting with the interview, is there anything that you want to tell me or ask me?

### Interview guide

1. Can you start by telling me about your point of view on precision medicine? (How would you describe it?)
   1. How does precision medicine affect your daily work? (your future work?)
2. What do you think are the pros and cons of precision medicine compared to traditional treatment?
3. What is your experience in discussing precision medicine with your patients?
   1. How did you experience patients' understanding of precision medicine?
4. In what way do you discuss treatment options with your patients? Develop (What do you address?)
5. In what way do you think that precision medicine and the experimental treatment options that are connected to precision medicine can influence how treatment options are discussed with patients?
6. How do you see the use of artificial intelligence and other heavy computational methods in oncology? Is this something ever discussed with patients?
7. Could the use of algorithms affect the relationship between patient and doctor?
   1. Do you think this raises questions about trust in doctors?
8. In what way can you imagine that the responsibility for treatment changes when one uses an algorithm?
   1. What consequences could that have?

That concludes the interview questions. I will now summarise our discussion

[Summary]

1. Would you like to clarify or add something?

## Appendix 2

### Mapping research focus to interview questions

Table S1. Mapping research focus to interview questions

| **Research Focus Area** | **Interview Questions** | **Insight Gained** |
| --- | --- | --- |
| Physicians’ general understanding and interpretation of precision medicine | Q1, Q2 | Helped identify how physicians conceptualise precision medicine and its practical and future implications |
| Effects of precision medicine on clinical routines | Q1a, Q2 | Revealed perceived benefits, challenges, and concerns about incorporating precision medicine in everyday care |
| Communication challenges around precision medicine | Q3, Q3a,  Q4, Q5 | Uncovered physicians' strategies, difficulties, and perceptions of patient comprehension |
| Role and perceived impact of AI and computational tools in oncology | Q6, Q7, Q7a | Explored how physicians perceive AI potential to support or interfere with care and the doctor-patient relationship |
| Responsibility and trust in AI-assisted decisions | Q7, Q8, Q8a | Provided insights into concerns about shifting responsibility and preserving trust in the clinical encounter |
| Ethical and professional implications of AI and precision medicine integration | Q5, Q6, Q7,  Q8, Q8a | Illuminated concerns about decision-making ethics, accountability, and professional autonomy |
